# Reproducibility of CT-based radiomic features against image resampling and perturbations for tumour and healthy kidney in renal cancer patients

**DOI:** 10.1038/s41598-021-90985-y

**Published:** 2021-06-02

**Authors:** Margherita Mottola, Stephan Ursprung, Leonardo Rundo, Lorena Escudero Sanchez, Tobias Klatte, Iosif Mendichovszky, Grant D Stewart, Evis Sala, Alessandro Bevilacqua

**Affiliations:** 1 Department of Electrical, Electronic, and Information Engineering (DEI), University of Bologna, 40136 Bologna, Italy; 2 Advanced Research Center on Electronic Systems (ARCES), University of Bologna, 40125 Bologna, Italy; 3 Department of Radiology, University of Cambridge, Cambridge, CB2 0QQ UK; 4 Cancer Research UK Cambridge Centre, University of Cambridge, Cambridge, CB2 0RE UK; 5 Department of Surgery, University of Cambridge, Cambridge, CB2 0QQ UK; 6 Department of Urology, Royal Bournemouth Hospital, Bournemouth, BH7 7DW UK; 7 Department of Computer Science and Engineering (DISI), University of Bologna, 40136 Bologna, Italy

**Keywords:** Biomarkers, Kidney, Biomedical engineering, Statistics

## Abstract

Computed Tomography (CT) is widely used in oncology for morphological evaluation and diagnosis, commonly through visual assessments, often exploiting semi-automatic tools as well. Well-established automatic methods for quantitative imaging offer the opportunity to enrich the radiologist interpretation with a large number of radiomic features, which need to be highly reproducible to be used reliably in clinical practice. This study investigates feature reproducibility against noise, varying resolutions and segmentations (achieved by perturbing the regions of interest), in a CT dataset with heterogeneous voxel size of 98 renal cell carcinomas (RCCs) and 93 contralateral normal kidneys (CK). In particular, first order (FO) and second order texture features based on both 2D and 3D grey level co-occurrence matrices (GLCMs) were considered. Moreover, this study carries out a comparative analysis of three of the most commonly used interpolation methods, which need to be selected before any resampling procedure. Results showed that the Lanczos interpolation is the most effective at preserving original information in resampling, where the median slice resolution coupled with the native slice spacing allows the best reproducibility, with 94.6% and 87.7% of features, in RCC and CK, respectively. GLCMs show their maximum reproducibility when used at short distances.

## Introduction

Computed Tomography (CT) is one of the most widely used technologies for morphological imaging and the standard of care adopted in oncology for diagnosis, staging and treatment follow-up. It is still based on visual lesion detection and morphological measurements (e.g. maximum diameter, size, etc.), often performed with the aid of software tools^1^. Nonetheless, since the early nineties^2^, radiologists have benefited from computer-aided systems exploiting a large number of features, developed by specialized research groups. The recent increase in high-performance computing resources in entry-level workstations and the growth of automatic tools for radiomic analyses, has made them popular and accessible to research groups. Accordingly, the number of radiomic studies has exploded, a large number of features is analysed to measure macroscopic tumour or tissue characteristics or to find latent properties^3^. The automatic quantification of tissue features based on radiomic approaches has shown improvements in terms of both reproducibility, discrimination and classification capability^4^, thus increasing the number of candidate imaging biomarkers^3^. However, the plethora of software packages available for radiomic analyses used by groups, with different degrees of expertise, has highlighted the urgent need for standardisation of methodology and measurements^5,6^. In fact, many factors are known to induce variability in radiomic features including noise^7^, heterogeneous voxel size^8^ and other CT parameter settings^9^, Region Of Interest (ROI) segmentation^10–13^, as well as tumour phenotype^14^. Despite its importance, only a few studies perform a dedicated analysis of the robustness and reproducibility of radiomic studies. Some of them use phantoms to explore the effects of variable acquisition parameters, such as tube current^7^, or voxel size^15^. Other radiomic studies assess the effects of varying segmentations on first or second order texture features, for instance, in non-small cell lung cancer (NSCLC)^1,16^, and rectal cancer^17^. A different approach has been recently proposed^18^, where the authors test different perturbation chains on NSCLC and head and neck cancer datasets, to find the chain better reproducing the outcome of a test-retest procedure, to be used when such method is not applicable. All these studies, carried out on different tumours, analyse the reproducibility mainly against varying ROI segmentation, or a set of perturbations.

To the best of our knowledge, this is the first work assessing robustness of first order (FO) and 2D and 3D second order texture features in CT imaging of renal cell carcinoma (RCC) and normal kidney (CK), by addressing three types of perturbations induced by Added White Gussian Noise (AWGN) (N), different voxel-size (V) and varying ROI (R). In addition, we perform a comparative analysis to select the best interpolation methods to be preliminarily applied, if needed, before any feature extraction procedure. Finally, results can provide practical operating guidelines to choose the proper voxel size in case of datasets with heterogeneous in-plane resolutions and to aggregate information derived from grey level (GL) co-occurrence matrices (GLCMs)^19^, thus improving standardisation of radiomic studies.

## Methods

### Patient images

This study included 98 patients with RCC imaged at a single institution. CT acquisition parameters are provided in Table 1. Images were acquired with Siemens SOMATOM Definition AS/AS+ CT scanners, with iterative reconstruction kernel I30f$\setminus$3. Scan resolution ranges from square voxel spacing $v_s=0.541$ mm to $v_s=0.957$ mm, with mean value equals to 0.740, and $v_z=5$ mm-slice thickness. Mean values and ranges of tube voltage and exposure were 109 [100,140] KVp and 166 [137,535] mAs, respectively. Automatic tube voltage selection (CARE kV) and current modulation (CARE Dose) were employed to optimize the dose to patients, resulting in a mean and range of 109 [100,140] KVp and 166 [137,535] mAs, respectively. Image series of the corticomedullary and nephrographic phase, acquired at 35 and 100 s after the administration of the intravenous contrast agent (Omnipaque 300 mg I/ml, GE Healthcare) were included for 28 and 70 patients, respectively. This retrospective study was approved by Health Research Autority (HRA), University of Cambridge and Cambridge Research and Development (R&D) department that waived the written informed consent. This study was conducted according to relevant guidelines and regulations.

**Table 1:** CT image acquisition parameters.

Parameter	CT
Number of scans	98
Scanners	Siemens SOMATOM AS/AS+
Tube voltage (kVp)	109 [100,140]
Exposure (mAs)	166 [137,535]
Reconstruction kernel	I30f$\setminus$3
Square voxel spacing ([*x,y*]-axes; mm)	0.740 [0.541,0.957]
Voxel spacing (*z*-axis; mm)	5
Image noise ($\sigma$; HU)	4 [2.9, 5.9]
SNR (dB)	42 [37, 45]

### Segmentation

ROIs of RCC and CK were semi-automatically outlined using the MICROSOFT RADIOMICS TOOL (Version 1.0.30558.1, project InnerEye, https://www.microsoft.com/en-us/research/project/medical-image-analysis/) by a medical doctor and clinical researcher with three years experience in renal imaging. The structures were segmented in all slices at the original scan resolution (RCC volume was 196 cm^3^, on average). Polygonal ROIs were exported as DICOM RTSTRUCT and imported in MATLAB (Version R2019b, The MathWorks Inc, Natick, Massachusetts, https://it.mathworks.com/products/matlab.html/) to generate binary segmentation masks for RCC and CK. In particular, CK and RCC were segmented on the first and last slice where they were visible and contours for every other slice were interpolated^20^. Manual corrections were applied to sub-optimally segmented slices, leading to an iterative re-calculation of the remaining interpolated slices. The segmentation of CK included the renal cortex and medulla but not the the collecting system and hilar fat.

### Image processing

**Figure 1:**
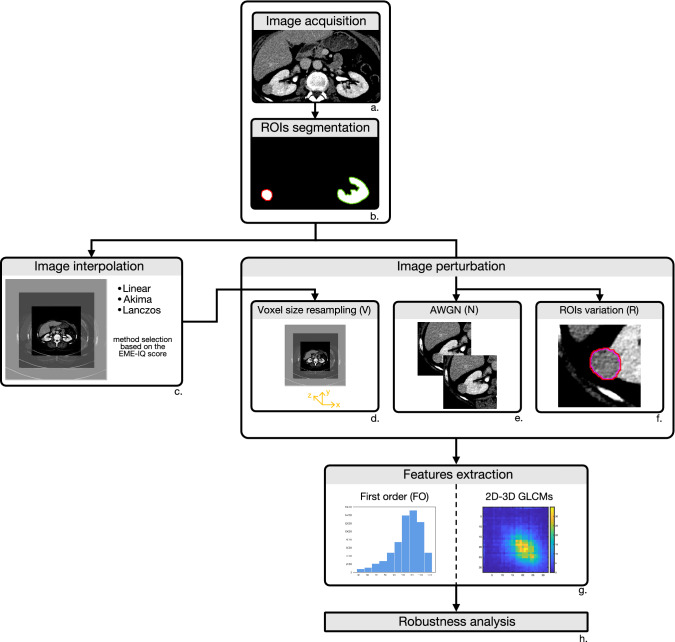
Workflow of CT image processing for feature robustness analysis. First, CT images were acquired (**a**) and ROIs segmented (**b**). Then, one out of three resampling methods was chosen based on the highest EME-IQ score (**c**), then CT images were perturbed by changing resolution (**d**), noise addition (**e**), and ROI variation (**f**). Finally, first order, and 2D and 3D texture features based on GLCMs (**g**) were extracted from original and perturbed images and robustness analysis was performed (**h**).

Image processing for feature robustness analysis was performed according to the workflow reported in Fig. 1, where the main steps are outlined. For each block of the flowchart more details are provided in the reference section of the main manuscript.

After CT image acquisition and segmentation, an image interpolation procedure was needed to standardize the different resolutions of the dataset. After discarding the highest and lowest 2.5% of grey values according to a simple and standard outlier removal procedure, three well-known interpolation methods were analysed and compared, to find the method best preserving the statistical properties of the original images, to be used for the image perturbation by voxel-size resampling. The comparison was carried out by exploiting the Enhancement Measurement Error-Image Quality (EME-IQ)^21^ score that measures local image contrast, amongst the most important information cues, so that higher EME-IQ values indicated the interpolation method better preserved sharpness and edges. After that, three different perturbations were applied to whole images and ROIs. The first perturbation considered was the AWGN, added to the original CT images using the same standard deviation ($\sigma$) as the original images. As the second perturbation, the original CT slices were resampled and interpolated along the *z* direction. Finally, to simulate inter-reader variability, ROI enlargement and shrinking were considered^18^. Lastly, 3D FO and 2D-3D texture features (from GLCMs) were computed on the original and perturbed CT images, and their reproducibility was studied. All the procedures were implemented in MATLAB.

### Image interpolation

Different scan voxel size is a well-known source of variability for radiomic features in a heterogeneous image dataset that should be taken into account when performing radiomic studies^8^. Hence, applying a resampling procedure is needed, to achieve one voxel size^15^. However, any resampling method relies on interpolation techniques that may potentially alter latent image properties, thus affecting the reproducibility of the features extracted from the resampled image and from the original^22^. In radiomic analyses, the interpolation method must be conceived for quantitative imaging and preserve the original properties of the CT GL distributions, rather than to simply yield visually pleasant images. In this work, we analysed and compared three well-known interpolation methods, that is, linear interpolation, largely employed in most radiomic studies^15^ thanks to its low complexity and computational cost, Akima cubic Hermite spline^23^ and Lanczos^24^ interpolation, the latter mostly used in computer vision and quantitative imaging. The comparison was performed by resampling the original CT images at the best and worst resolutions, corresponding to the smallest ($v_s^s=0.541$) and largest ($v_s^l=0.957$) original voxel size, and rounding the grey levels to the nearest integer. When employed for visualization purposes, the different interpolation methods are assessed through a forward-backward process, which compares the quality of original and restored image^25^. Here, we measured directly the quality of the interpolated images and adopted the EME-IQ score, a Non-Reference IQ measure that quantifies the level of local contrast^21^. For each patient, EME-IQ scores were computed and averaged on all CT slices, in both upsampling and downsampling. Then, the three interpolation methods were ranked according to their EME-IQ score. We chose the method that resulted in the best score for the highest number of patients for both upsampling and downsampling, and adopted it in the subsequent steps of V image perturbation.

### Image perturbation

#### Additive noise (AWGN)

CT images are known to be mostly affected by quantum noise, arising from the effects of the variability of electronic density of tissue voxels^26^, statistically represented by a random Gaussian process^27^. Therefore, we perturbed CT images by AWGN where, for each patient, $\sigma$ is given by the average of the standard deviation of each slice, estimated according to the method proposed by Ikeda et al.^28^.

#### Changing voxel size

Original CT images consisted of anisotropic voxels, with different in-plane resolutions, but one slice spacing that was on average one order of magnitude bigger. As regards slice resolution, we investigated three different resampling strategies, that are: (1) upsampling the whole dataset to $v_s^s$, (2) downsampling to $v_s^l$, (3) resampling at the median resolution ($v_s^M=0.741$ mm). Although working with isotropic voxels would be advisable, resampling to the $z$-axis resolution for isotropy would introduce an unrecoverable signal loss. Therefore, besides keeping the original scan resolution ($v_z=5$ mm), we limited the highest resolution to $v_z=1$ mm, exploring intermediate values, with 1-mm steps. In total, combining three voxel sizes with five slice thicknesses, we tested 15 different voxel sizes.

#### Segmentation perturbation

One of the causes affecting the clinical reliability of radiomic features as predictive or prognostic biomarkers is the lack of reproducibility of quantitative measurements, depending on the variability of intra- and inter-observer ROI segmentation^1^. Similarly to what done in^29^ and^18^, we simulated such variability, considering volume variations up to 20%, by ROI enlargement and shrinking. Actually, while ROI erosion just implies missing some tissue of the same type, ROI dilatation means including different tissues. Consequently, ROIs were shrunk by 10%, 15% and 20% or dilated by 10%. This procedure was carried out through binary morphological dilation and erosion, with a 3 × 3 pixel square structuring element (SE), according to a pixel-based random contourization procedure. Of course, it is unlikely to achieve the exact percentage variation, therefore the exceeding pixels were randomly removed to attain the expected percentage.

### Feature extraction

Radiomic features commonly employed to depict tumour heterogeneity can be grouped into first, second, and higher order statistical descriptors. In particular, FO features measure the statistical properties of the GL distribution and ignore its spatial relationships within the ROIs, whilst texture features (i.e. second and higher order ones) investigate the relationships between neighbouring grey levels at pixel- or region-level^30^. FO and GLCM-based texture features are very attractive, also because of their low computational complexity, they are computed in all radiomic packages^31^ and also employed for building predictive models in renal diseases^4^. In this study, we included 13 FO and 19 GLCM features computed in both 2D (GLCM2D) and 3D (GLCM3D), since no agreement exists yet on how to aggregate GLCM information to extract single representative features^6^. Hence, GLCM2D were computed in four directions, $\theta ={0}^\circ$, ${45}^\circ$, ${90}^\circ$, ${135}^\circ$, and GLCM3D were extended in 13 directions^32^, with five odd distances, from $\delta =1$ to $\delta =9$. The features were extracted after intensity-based outlier removal was performed on CT images at the $2.5\%$ threshold at both left and right tails of GL distributions. Based on a preliminary analysis of our CT dataset, the commonly used choice^17,33^ of 32 quantization levels was adopted for the GLCM computation. GLCMs were also symmetrized and direction-weighted. In GLCM2D, features were first computed on each slice and then averaged. In all, 108 radiomic features were computed on the original and perturbed CT images, for RCC and CK separately.

**Table 2:** List of first order (n=13) and GLCM2D-3D (n=19) features.

First order	GLCM2D-3D
Mean (*m*)	Autocorrelation (*autoc*)
Median (*M*)	Correlation (*corr*)
Skewness (*s*)	Cluster prominence (*cprom*)
Maximum value (*max*)	Homogeneity (*homom*)
*m* of last decile (*m90th*)	Maximum probability (*maxpr*)
*M* of last decile (*M90th*)	Contrast (*contr*)
Standard deviation (*std*)	Cluster shade (*cshade*)
*M* absolute deviation (*MAD*)	Variance (*sosvh*)
Interquartile range (*iqr*)	Dissimilarity (*dissi*)
Local coefficient of variation (*lCV*)	Energy (*energ*)
Uniformity (*u*)	Entropy (*entro*)
Entropy (*e*)	Difference variance (*dvarh*)
Kurtosis (*k*)	Difference entropy (*denth*)
	Information measure of *corr* (*inf1h*)
	Inverse difference normalized (*indnc*)
	Inverse difference moment normalized (*idmnc*)
	Sum average (*savgh*)
	Sum variance (*svarh*)
	Sum *entro* (*senth*)

Table 2 lists all FO and GLCM features, while a detailed mathematical formulation of the radiomic features extracted is also provided in Supplementary Note 1.

### Robustness analysis

All the extracted features were analysed for both RCC and CK and robustness was assessed using the Intraclass Correlation Coefficient (ICC)(1,1) with 95$\%$ confidence interval (CI)^18^. Radiomic features were considered as being robust (*r*) if ICC 95$\%$ CI $\ge$ 0.90, non-robust (*nr*) if CI < 0.90, and with indeterminate robustness (*i*) otherwise (i.e. with 0.90 strictly included in CI). In total, 29 perturbations were assessed, one arising from N, 24 combinations of V, and four from R, as detailed in Table 3.

First, the robustness of all radiomic features together was investigated against all perturbations, to have an overview of features behaviour depending on the tissue phenotype (i.e. RCC or CK) only. Mean percentage of *r*, *nr*, and *i* features were reported for each perturbation type. Moreover, the proportional contribution given by each feature class to the global robustness was investigated, together with the contribution of the single features. In practice, robustness was assessed (i) for all feature classes (i.e. FO, GLCM2D, GLCM3D) against all image perturbations, (ii) for each feature class against each perturbation type (i.e. N, V, R), and (iii) for each feature against all image perturbations.

**Table 3:** Descriptions of the 29 perturbations assessed.

Number	Type	Perturbation	Description
1	N	N	AWGN
15	V	$v_s-v_z$	15 voxel-sizes by combining $v_s^s$, $v_s^M$, $v_s^l$ with $v_z$ in [1, 5]
1		V	Global assessment of the all 15 voxel-sizes
1		$v_s^s$	Resolution $v_s^s$ kept fixed and $v_z$ in [1, 5]
1		$v_s^M$	Resolution $v_s^M$ kept fixed and $v_z$ in [1, 5]
1		$v_s^l$	Resolution $v_s^l$ kept fixed and $v_z$ in [1, 5]
1		Z1	Resolution $v_z=1$ kept fixed for all $v_s^s$, $v_s^M$, $v_s^l$
1		Z2	Resolution $v_z=2$ kept fixed for all $v_s^s$, $v_s^M$, $v_s^l$
1		Z3	Resolution $v_z=3$ kept fixed for all $v_s^s$, $v_s^M$, $v_s^l$
1		Z4	Resolution $v_z=4$ kept fixed for all $v_s^s$, $v_s^M$, $v_s^l$
1		Z5	Resolution $v_z=5$ kept fixed for all $v_s^s$, $v_s^M$, $v_s^l$
4	R	R+10	Dilation, volume variation equals to +10%
		R-10	Erosion, volume variation equals to -10%
		R-15	Erosion, volume variation equals to -15%
		R-20	Erosion, volume variation equals to -20%

Finally, this study assessed the real need for having GLCMs computed at multiple $\delta$ distances, because of their known high correlation. To this end, we performed this analysis by adopting the voxel size resulting as the most reliable from analyses at step (i). In conclusion, the correlation of features computed at all $\delta$ was measured through the linear Pearson coefficient ($\rho$) and the statistical significance of the differences was assessed by the ANOVA test (*p*-value $\le$ 0.001).

## Results

### Interpolation methods

**Figure 2:**
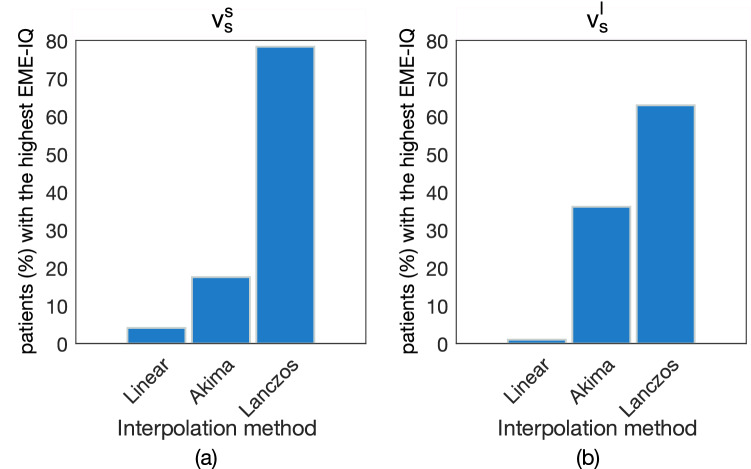
Comparison of linear, Akima, and Lanczos interpolation methods, based on the percentage of patients receiving the highest EME-IQ score, for both upsampling (**a**) and downsampling (**b**) respectively at the smallest ($v_s^s$) and the largest ($v_s^l$) resolutions of the CT image dataset. The Lanczos method yielded the highest EME-IQ score in 78$\%$ and 63$\%$ of patients, respectively.

Figure 2 reports the comparison of Linear, Akima and Lanczos interpolation methods based on the EME-IQ score. When resampling at $v_s^s$ (Fig. 2a), the linear method achieved the best result for only 4% of patients, Akima for 18% and Lanczos for 78% of patients. This ranking was confirmed when resampling at $v_s^l$ (Fig. 2b), where linear and Akima methods reached 1% and 36%, respectively, whilst Lanczos still proved to be the best one for 63$\%$ of patients. On the original CT images, mean EME-IQ score was 2.87, ranging between [1.36,6.72], whilst on upsampled images, mean and range EME-IQ values increased, being 2.99 [1.61,6.48] for linear interpolation, 3.09 [1.68,6.71] for Akima, and 3.12 [1.70,6.80] for Lanczos method. When downsampling, EME-IQ scores decreased with respect to the original CT images, with 2.71 [1.53,5.40] for linear, 2.84 [1.63, 5.66] for Akima, and 2.85 [1.65, 5.64] for Lanczos. To allows readers to assess the visual differences of these three methods, some exemplifying images are shown in Supplementary Figure S1 for three patients where linear, Akima, and Lanczos methods were respectively the best methods, in either upsampling ($v_s^s$) and downsampling ($v_s^l$), or both. Therefore, hereafter Lanczos is chosen as the reference interpolation method.

### Robustness of feature classes against image perturbation

**Figure 3:**
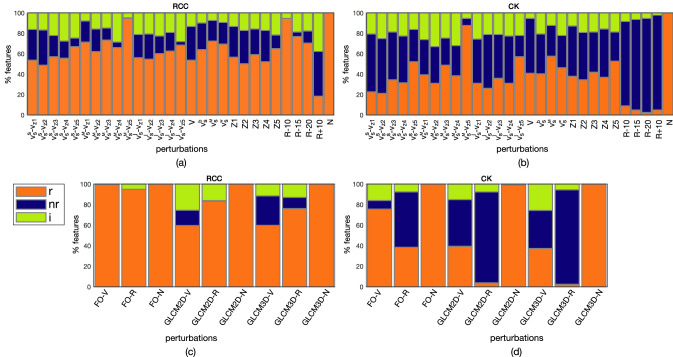
Overall percentage of robust (*r*), non-robust (*nr*), and with indeterminate robustness (*i*) features against image perturbation for RCC (**a**) and CK (**b**), separately. Feature robustness is also reported per each feature class (i.e. FO, GLCM2D, and GLCM3D) for RCC (**c**) and CK (**d**).

Figure 3a,b reports the percentage of *r*, *nr*, and *i* features against each perturbation for RCC (Fig. 3a) and CK (Fig. 3b), respectively (detailed robustness performance of each feature class against all image perturbations are reported in Supplementary Figure S2). Summarizing the information of Fig. 3a, in RCC there are, on average against all perturbations, 65.6% of *r* features, 18.0% *nr*, and 16.4% *i*. Similarly in Fig. 3b, in CK 39.0% was *r*, 42.9% *nr*, the remaining 18.0% *i*. In both RCC and CK, the highest percentage of *r* features is achieved with the N perturbation (last columns of Fig. 3a,b), where practically all features were *r* (*r*-RCC: 100%, *r*-CK: 99.6%), or at worst *i* (*i*-CK: 0.4%). As regards V perturbations, whilst in RCC the percentage of *r* and *nr* features was 73.0% and 14.5%, in CK values are lower, with *r*-CK: 50.8% and *nr*-CK: 30.3%. Despite this difference, RCC and CK showed a common behaviour against all perturbations at fixed $v_z$ values (i.e. [Z1–Z5]), both having a low percentage of *r* features (on average, 56.7% for RCC and 40.8% for CK) if compared with those at fixed $v_s$ resolutions (i.e. $v_s^s$, $v_s^M$, $v_s^l$), that is on average 68.6% for RCC and 48.1% for CK. In addition, if considering $v_z$, most of the 15 combinations had more than 60% of *r* features in RCC, mainly referred to $v_s^M$ coupled with multiple $v_z$ values, with the highest percentage (94.6%) achieved with $v_s^M$-Z5, that is, without interpolating along the *z*-direction. This couple was also the best in CK, with 87.7% of *r* features. As regards R perturbations, whilst in RCC there was 94.6%, 76.9%, and 70.4% of *r* features at R-10, R-15, and R-20, respectively, in CK they were 8.9%, 4.9%, 2.5%, respectively. Finally, as regards R+10, RCC showed 19% of *r* features, while CK had only 4% of them.

Figure 3c,d focuses on feature robustness of each feature class per perturbation type (values are also reported in Table 4).

**Table 4:** Feature robustness against image perturbations referred to Fig. 3c,d.

	RCC									CK								
	FO			GLCM2D			GLCM3D			FO			GLCM2D			GLCM3D		
	N	V	R	N	V	R	N	V	R	N	V	R	N	V	R	N	V	R
*r* (%)	100	100	94.9	100	59.7	83.2	100	59.8	76.1	100	75.6	38.5	98.9	39.4	3.9	100	37.3	2.5
*nr* (%)	0	0	0	0	14.9	0.7	0	28.7	10.9	0	8.3	53.8	0	45.4	88.4	0	37.0	91.9
*i* (%)	0	0	5.1	0	25.4	16.1	0	11.5	13	0	16.1	7.7	1.1	15.2	7.7	0	25.7	5.6

FO features hold the highest percentage of *r* features, in both RCC and CK, for V (RCC: 100.0%, CK: 75.6%), R (RCC: 94.9%, CK: 38.5%), and N (RCC and CK: 100.0%) perturbation types. GLCM2D and GLCM3D features achieved comparable results in both RCC and CK. In fact, the percentage of *r* features averaged over all perturbations was GLCM2D: 80.9% and GLCM3D: 78.6% in RCC, and GLCM2D: 47.4% and GLCM3D: 46.4% in CK. In particular, robustness against V and R was always higher in RCC than in CK. In particular, R perturbation showed the greatest difference, with GLCM2D-R: 83.2% and GLCM3D-R: 76.1% in RCC and GLCM2D-R: 3.9% and GLCM3D-R: 2.5% in CK. Finally, analysing how each of the 29 perturbations affected each feature class (Supplementary Figure S2), one can see that while for CK there were no differences between GLCM2D and GLCM3D (the same 3 perturbations showed at least 60% of *r* features), in RCC 13 and 10 perturbations showed at least 60% of *r* features, in GLCM2D and GLCM3D, respectively, but only 7 of them were shared.

### Robustness analysis of single features

All the FO features were *r* features in RCC in at least $60\%$ of perturbations, and 9 of them (*m*, *M*, *max*, *m90th*, *M90th*, *iqr*, *u*, *e*, and *lCV*) were confirmed in CK too. As far as second order features are concerned, 48 GLCM2D and 53 GLCM3D resulted *r* features in RCC in at least $60\%$ of perturbations, and 44 of them were in both classes. In CK, 28 GLCM2D and 24 GLCM3D resulted *r* features in at least 60% of perturbations and 21 of them were shared. Finally, as regards *r* features shared between RCC and CK, they were *autoc*, *entro*, *savgh*, *sentro*, *sosvh*, *svarh*. It is worth mentioning that *cprom*, *cshad*, *energ*, *inf1h*, and *maxpr* were found in RCC only.

**Figure 4:**
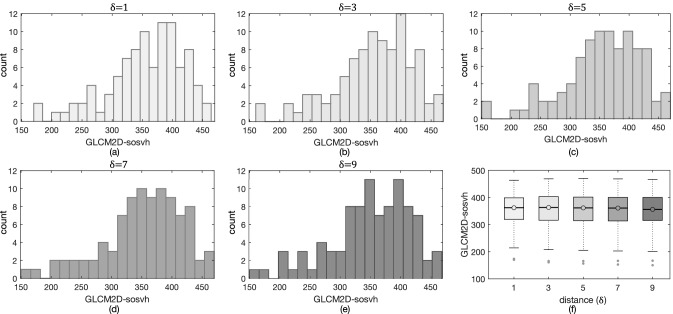
Histograms of GLCM2D-*sosvh* computed from $\delta =1$ to $\delta =9$ (**a**–**e**) did not show any relevant difference. In addition, boxplots (**f**) confirmed no statistical significance at ANOVA test (*p*-value=0.97).

GLCM features have an intrinsic redundancy, since they were computed at multiple $\delta$, and these measures are often highly correlated. This is true also in this study, with $\rho \ge 0.90$ for all the selected features and ANOVA tests yielding *p*-values$~>0.03$ for almost all $\delta$, that is far above the established significance threshold, meaning they perform the same. For exemplification purposes, we show in Fig. 4a–e histograms for a representative feature, GLCM2D-*sosvh*, computed in RCC at multiple $\delta$, where no relevant difference in distributions can be detected. This becomes more explicit in Fig. 4f, showing the boxplots of GLCM2D-*sosvh* for all $\delta$, where ANOVA test confirmed their statistically equivalence (*p*-value$~=0.97$). Actually, the equivalence between distances weakens as they shorten. For instance, the features *entro* and *sentro* representing local tissue heterogeneity showed significant differences between $\delta =1$ and $\delta =3$ in both RCC and CK (*p*-value$~\le 10^{-6}$), and between $\delta =3$ and $\delta =5$ in CK only. Analogously, the features *cprom*, *maxpr*, and *inf1h* in RCC were statistically equivalent for $\delta \ge 3$, and different from $\delta =1$ (*p*-value$~\le 10^{-4}$).

## Discussion

Assessing the robustness of radiomic features has become necessary to determine feature reproducibility before translating predictive and prognostic radiomic biomarkers into clinical practice. Feature robustness depends on the tumour phenotype and is not generalizable^14^, hence, this study focuses on the need for analysing feature robustness on RCC in CT using one of the largest datasets^22^. In addition, the analysis is extended to CK to determine which features might be robust enough to assess, for instance, diffuse renal diseases. In particular, in this study we analyse the robustness of radiomic features against some of the most frequent sources of variability, which are noise, heterogeneous scan voxel size and varying segmentation. Moreover, this study offers an in-depth analysis of three known interpolation methods aiming at supporting researchers in choosing the most appropriate one when resampling CT images. Results show that Lanczos interpolation outperforms the other methods in both upsampling and downsampling procedures. In particular, this study highlights one of the major limits of linear interpolation, probably the method most widely employed in radiomic studies for feature stability assessment in heterogeneous datasets and suggested by the Imaging Biomarker Standardization Initiative (IBSI)^6^. More specifically, if on the one hand resampling based on linear interpolation improves visual images’ perception, on the other hand it smooths tissue edges and texture variation, thus limiting quantitative information^34^. Some authors are aware of the importance of the interpolation methods, which may influence feature robustness. For instance, Whybra et al.^30^ carried out a comparison of feature robustness, after linear and spline interpolations. The authors concluded that the two methods were equivalent since no difference existed in terms of feature stability, albeit in the presence of large numerical variations. However, although those features may be reproducible, this does not ensure that the features are correctly representing the original CT image information. For this purpose, we recommend a preliminary analysis to assess that the resampling procedure does not affect the properties of the GLs distribution. It is worth noting that the upsampling procedure, although adding artificial information, improves the original image quality (EME-IQ = 2.87) with all methods considered (EME-IQ = 2.99 for linear, EME-IQ = 3.09 for Akima, and EME-IQ = 3.12 for Lanczos). Even more relevant, when downsampling, while linear interpolation degrades (EME-IQ = 2.71) original image quality, Lanczos, performing the best (EME-IQ = 2.85), preserves the EME-IQ score of the original image.

With regard to feature robustness, there are many more *r* features in RCC (65.6%) than in CK (39.0%), although both RCC and CK show an excellent robustness against N perturbation (N-RCC: $100\%$, N-CK: $99.6\%$). This agrees with the outcome of Zwanenburg et al.^18^, which similarly found that the highest percentage of robust features was for N perturbations. Instead, substantial differences of *r* features between RCC and CK are found under R and V perturbations. In fact, results show that against R- perturbations, while in RCC *r* features are never lower than 70%, in CK a very low percentage of features are reproducible if changes in volumes are higher than 10%. As expected, *r* features have a much worse performance against R+10 perturbation, this suggesting that when segmenting it is always better performing a “safe” contouring, that is, underestimating rather than overestimating the ROI.

When resampling a heterogeneous CT dataset, the goal is to minimize interpolation artefacts. Our results show that choosing the median resolution ($v_s^M$) does this, with a greater effect in CK rather than in RCC. In fact, among the different CT voxel sizes, $v_s^M$ achieves the highest percentage of *r* features, this suggests that resampling at the median voxel size is strongly recommended. In addition, focussing on the different $v_s$-$v_z$ couples, $v_s^M$ performs best when no interpolation in the *z*-direction is carried out between slices. This is somewhat expected, since the large difference between $v_s$ (higher) and $v_z$ (lower) voxel sizes makes the interpolation along the *z*-direction introduce a low reliable signal, if compared with the information in the original CT slices. Accordingly, while resampling along *z*-axis should be carefully evaluated, especially in case of a large slice spacing, preservation of the original $v_z$ resolution could be in most cases the best choice.

Our robustness analysis finds the FO features are definitely the most reproducible ones, confirming what was reported in the review of the most recent research works regarding feature repeatability and reproducibility by Traverso et al.^22^. In addition, all *r* features in CK (9/13) are robust in RCC as well. Besides the well-established statistical descriptors (e.g. *m*, *M*, etc.), there are both *lCV* and *e*, two common indicators for measuring local heterogeneity or irregularity, that is also one of the changing properties of normal tissues while shifting into tumour ones^35^. The remaining 4/13 FO features resulted robust in RCC only, thus showing a higher specificity for tumour tissues, which could be useful for specific tumour-related clinical questions. It is worth noting that also all GLCM features (both 2D and 3D) that prove to be robust in CK, are robust in RCC too, while other features, measuring local asymmetries of GLCMs, are more tumour-specific.

This research also investigates the well-known phenomenon of the high correlation of GLCM-based texture features computed at different distances, to see whether and to what extent using higher distances is worth. In practice, almost all features are shown to be equivalent when computed at distances from 3 to 9. Five *r* features show a difference in RCC when computed at $\delta =1$ and $\delta =3$ and two only in CK at $\delta =3$ and $\delta \ge 5$. This evidence is yet more relevant if considering that even in CK, having really wide ROIs, distances higher than $\delta =3$ are most of times equivalent. This could suggest that computing textures at distance $\delta =3$ should be general enough, thus allowing feature selection to be simpler and more effective, besides reducing computational burden. As a general remark, the recent literature lacks comparative studies between 2D and 3D texture feature robustness, and even when features are compared on the basis of their capabilities (e.g., predictive ability, and so on) the outcomes are controversial^36^. Our results show that the overall robustness of GLCM features computed in 2D or 3D is similar. However, our findings show a higher number of *r* features for GLCM2D and, at the same time, a higher number of perturbations not affecting robustness of GLCM2D features. Therefore, GLCM2D texture features should be preferred.

The main limitation of this study is that only first and second order features are considered. However, these are the first features whose robustness has been analysed in radiomic studies based on CT images of renal disease. In addition, our methodological approach can be exploited to extend the study to include more feature classes. An additional limitation arises from having considered 10% as the lowest volume variation bound. This threshold might yield overestimated inaccuracies of radiologist’s segmentations. Therefore, the results reported in this study can be considered as the worst scenario. Finally, another limitation is the analysis of all RCC subtypes together. However, an analysis of feature robustness across different RCC subtypes would have been beyond the scope of this study.

This work aimed at assessing the robustness of radiomic features against some of the most common sources of variability. Our findings allowed drawing some concluding remarks that could be useful guidelines for radiomic studies. In particular, texture features should be used at very short distances, heterogeneous CT datasets have to be resampled at the median slice resolution, whilst should not be interpolated along the cranial-caudal direction and, Lanczos should be used as the interpolation method.

## Supplementary Information

Supplementary Information.
