# Supplementary material for: Reproducibility of CT-based radiomic features against image resampling and perturbations for tumour and healthy kidney in renal cancer patients
Source: Sci Rep. 2021 Jun 2;11:11542. doi: 10.1038/s41598-021-90985-y (PMC8172898; doi:10.1038/s41598-021-90985-y)
Supplement: Supplementary file 1 — Supplementary Information. [file 41598_2021_90985_MOESM1_ESM.pdf]

Reproducibility of CT-based radiomic features against image resampling and perturbations for tumour and healthy kidney in renal cancer patients

Supplementary Materials

Margherita Mottola, Stephan Ursprung, Leonardo Rundo, Lorena Escudero Sanchez,
Tobias Klatte, Iosif Mendichovszky, Grant D Stewart, Evis Sala, Alessandro Bevilacqua

Supplementary Note 1: mathematical formulation of features

First order (FO)

Let Ω be the domain where the features were computed on, that is, the ROIs of RCC and CK computed over all slices, and N the number of pixels in Ω. Let also p_{x_i} be the probability of each pixel value $x_i \in \Omega$.

$x_i \in \Omega$, $i = 1...N$

$$\text{mean, } m = \frac{1}{N}\sum_i x_i \tag{1}$$

$$\text{median, } M = x_{\left[\frac{fc}{2}\right]}, fc = \frac{\sum_i N p_{x_i}}{2} \tag{2}$$

$$\text{skewness, } s = \frac{\frac{1}{N}\sum_i (x_i - \bar{x})^3}{\left(\frac{1}{N}\sum_i (x_i - \bar{x})^2\right)^{\frac{3}{2}}} \tag{3}$$

$$\text{maximum value, } max = \max_{x_i}(\Omega) \tag{4}$$

$$m\text{of last decile, } m90th = m(x \in x_i \geq x_{90th}) \tag{5}$$

$$M\text{of last decile, } M90th = M(x \in x_i \geq x_{90th}) \tag{6}$$

$$\text{standard deviation, } std = \sqrt{\frac{\sum_i (x_i - \bar{x})^2}{N}} \tag{7}$$

$$M\text{absolute deviation, } MAD = M(|x_i - M(\Omega)|) \tag{8}$$

$$\text{interquartile range, } iqr = M_{upper\ half} - M_{lower\ half} \tag{9}$$

$$\text{local coefficient of variation, } lCV = \frac{\sqrt{\frac{\sum_i (x_i - \bar{x})^2}{N}}}{\frac{1}{N}\sum_i x_i} \tag{10}$$

$$\text{uniformity, } u = \sum p_{x_i}^2 \tag{11}$$

$$\text{entropy, } e = -\sum_i p_{x_i} log_2 (p_{x_i}) \tag{12}$$

$$\text{kurtosis, } k = \frac{\frac{1}{N}\sum_i (x_i - \bar{x})^4}{\left(\frac{1}{N}\sum_i (x_i - \bar{x})^2\right)^2} - 3 \tag{13}$$

Second order texture features based on GLCMs

Let $q(i,j)$ be the $(i,j)-th$ entry in the GLCM quantized in N_g levels. Features' formulation is reported below according to the following notations:

$$\left.\begin{array}{l} q_x(i) = \sum_j q(i,j) \\ q_y(j) = \sum_i q(i,j) \end{array}\right\} \text{equal for symmetric GLCMs} \tag{14}$$

$$\left.\begin{array}{l} \mu_x = \sum_i \sum_j i \cdot q(i,j) \\ \mu_y = \sum_i \sum_j j \cdot q(i,j) \end{array}\right\} \mu, \text{ equal for symmetric GLCMs} \tag{15}$$

$$\left.\begin{array}{l} \sigma_x = \sum_i \sum_j (i-\mu_x)^2 \cdot q(i,j) \\ \sigma_y = \sum_i \sum_j (j-\mu_y)^2 \cdot q(i,j) \end{array}\right\} \sigma, \text{ equal for symmetric GLCMs} \tag{16}$$

$$q_{x+y}(k) = \sum_i \sum_j q(i,j)|_{i+j=k}, \quad \text{where } k = 2,3,...,2N_g \tag{17}$$

$$q_{x-y}(k) = \sum_i \sum_j q(i,j)|_{|i-j|=k}, \quad \text{where } k = 0,1,...,N_g-1 \tag{18}$$

$$\left.\begin{array}{l} HX = -\sum_i q_x(i) \cdot log q_x(i) \\ HY = -\sum_i q_y(i) \cdot log q_y(i) \end{array}\right\} \text{equal for symmetric GLCMs} \tag{19}$$

$$HXY = -\sum_i q(i,j) \cdot log(q(i,j)) \tag{20}$$

$$HXY1 = -\sum_i \sum_j q(i,j) \cdot log\left[q_x(i) \cdot q_y(j)\right] \tag{21}$$

Then, the features are as follows:

$$\text{autocorrelation, } autoc = \sum_i \sum_j (i,j) \cdot q(i,j) \tag{22}$$

$$\text{correlation, } corr = \frac{\sum_i \sum_j (i,j) q(i,j) - \mu_x \mu_y}{\sigma_x \sigma_y} \tag{23}$$

$$\text{cluster prominence, } cprom = \sum_i \sum_j (i+j-\mu_x-\mu_y)^4 \cdot q(i,j) \tag{24}$$

$$\text{homogeneity, } homom = \sum_i \sum_j \frac{q(i,j)}{1+(i-j)^2} \tag{25}$$

$$\text{maximum probability, } maxpr = \max_{i,j} q(i,j) \tag{26}$$

$$\text{contrast, } contr = \sum_i \sum_j (i-j)^2 q(i,j) \tag{27}$$

cluster shade, $cshade = \sum_i \sum_j (i + j - \mu_x - \mu_y)^3 \cdot q(i,j)$ (28)

variance, $sosvh = \sum_i \sum_j (i - \mu)^2 q(i,j)$ (29)

dissimilarity, $dissi = \sum_i \sum_j |i - j| \cdot q(i,j)$ (30)

energy, $energ = \sum_i \sum_j q(i,j)^2$ (31)

entropy, $entro = -\sum_i \sum_j q(i,j) \log_2(q(i,j))$ (32)

$$\text{difference entropy, } denth = -\sum_{k=0}^{N_g-1} q_{x-y}(k) log(p_{x-y}(k)) \tag{33}$$

$$\text{difference variance, } dvarh = -\sum_{k=0}^{N_g-1} (k - \mu_{x-y})^2 \cdot q_{x-y}(k) \tag{34}$$

$$\text{information measure of } corr, \; inf1h = \frac{HXY - HXY1}{\max(HX, HY)} \tag{35}$$

$$\text{inverse difference normalized, } indnc = \sum_i \sum_j \frac{q(i,j)}{1 + \frac{|i-j|}{N_g}} \tag{36}$$

$$\text{inverse difference moment normalized, } idmnc = \sum_i \sum_j \frac{q(i,j)}{1 + \frac{|i-j|^2}{N_g{}^2}} \tag{37}$$

$$\text{sum average, } savgh = \sum_{k=2}^{2N_g} k \cdot q_{x+y}(k) \tag{38}$$

$$\text{sum variance, } svarh = \sum_{k=2}^{2N_g} (k - \mu_{x+y})^2 \cdot q_{x+y}(k) \tag{39}$$

$$\text{sum } entro, senth = -\sum_{k=2}^{2N_g} q_{x+y}(k) \cdot log(p_{x+y}(k)) \tag{40}$$

Supplementary Figure S1: comparison of interpolation methods

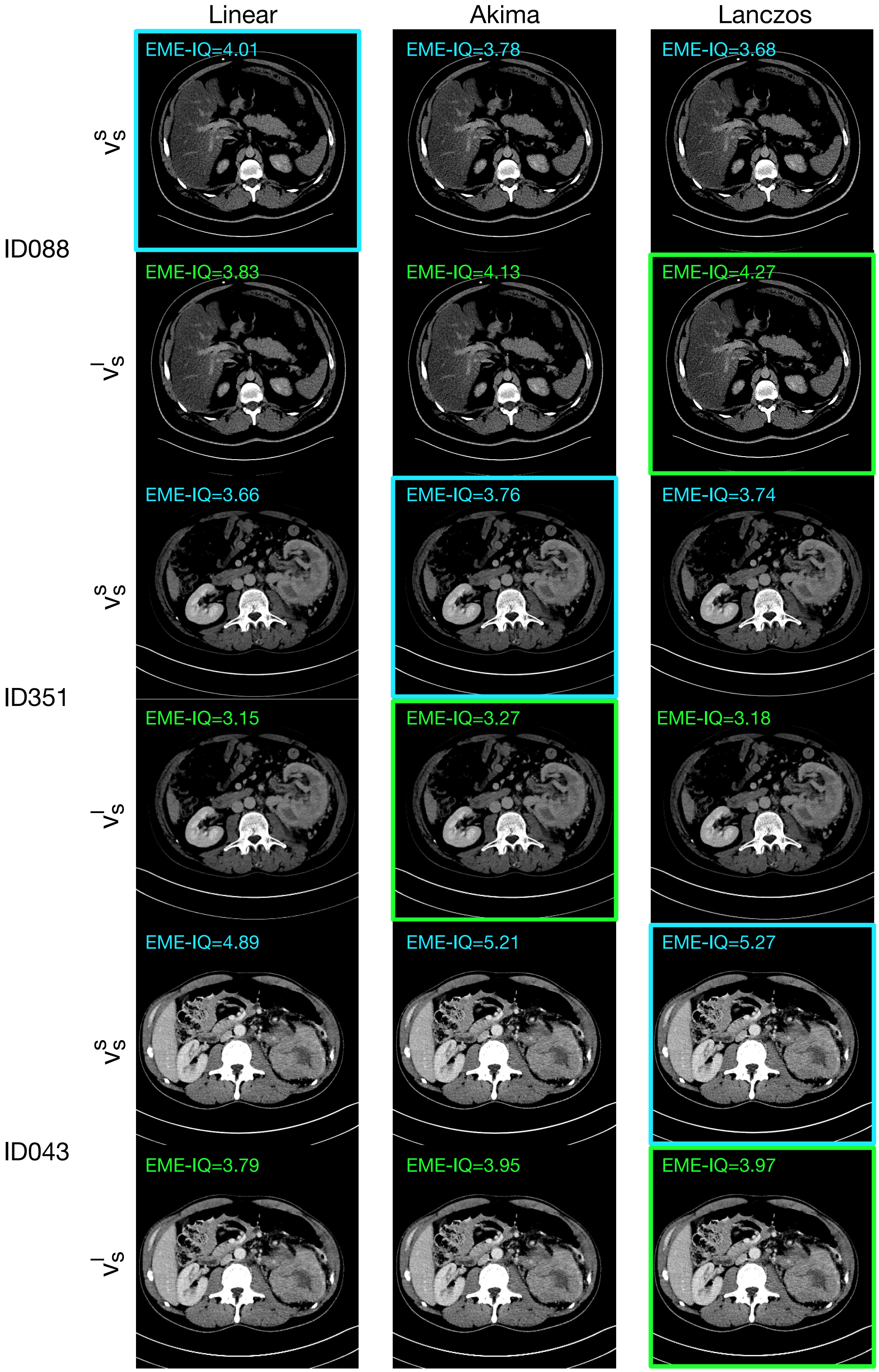

Figure 1. For patients ID088, ID351, and ID043, CT images resampled at v_s^s and v_s^l with Linear (left), Akima (centre), and Lanczos (right) interpolation methods. The EME-IQ scores of each image are reported and the best results are highlighted in upsampling (light blue) and downsampling (light green). For each v_s, the best image is highlighted with a coloured square

Supplementary Figure S2: robustness of each feature class

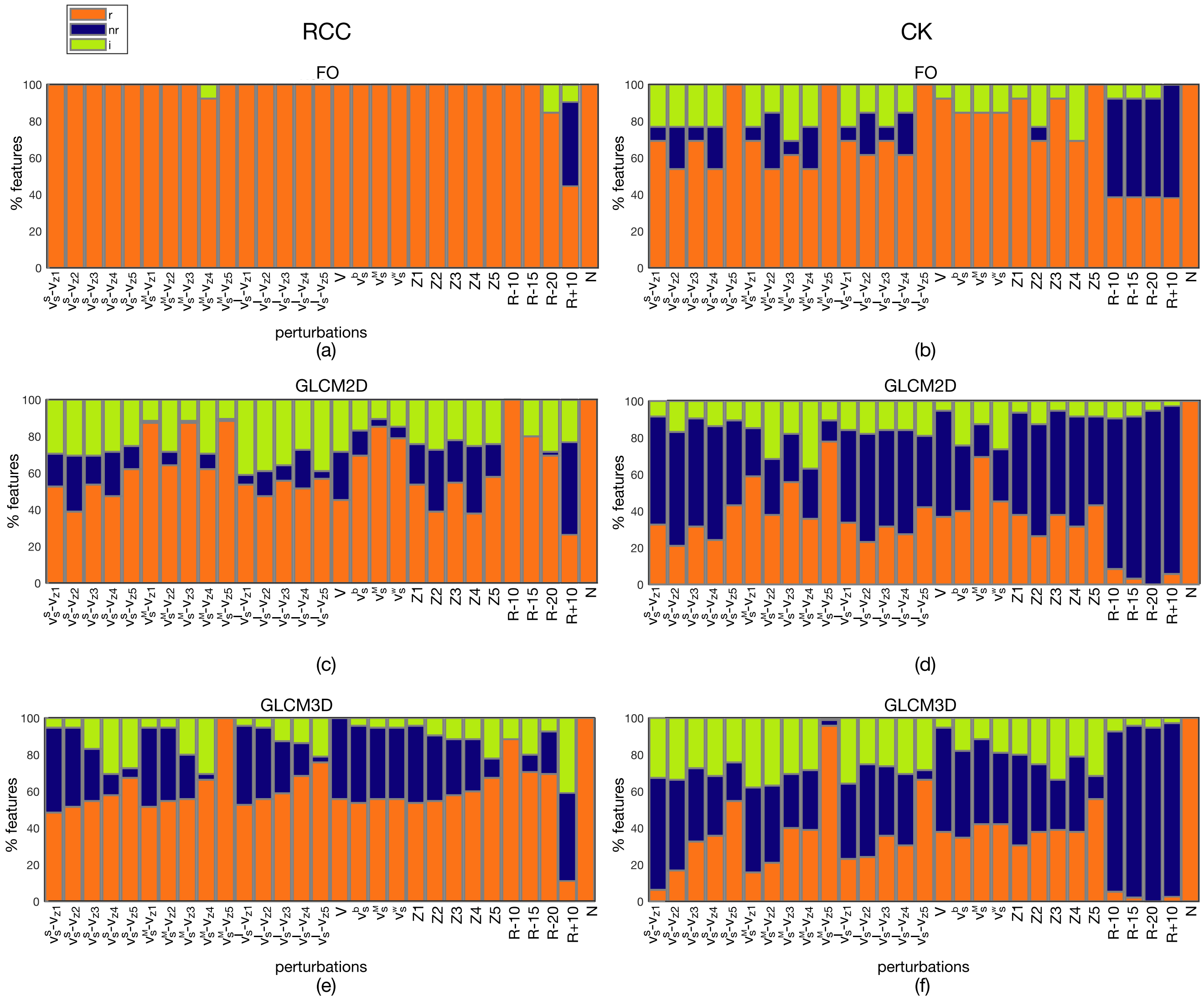

Figure 2. Robustness of each feature class, FO (a,b), GLCM2D (c,d), and GLCM3D (e,f) against all the 29 perturbations, for RCC (a,c,e) and CK (b,d,f), respectively.
